# Massive health education through technological mediation: Analyses and impacts on the syphilis epidemic in Brazil

**DOI:** 10.3389/fpubh.2022.944213

**Published:** 2022-09-27

**Authors:** Alexandre R. Caitano, Cristine M. G. Gusmão, Sara Dias-Trindade, Ingridy M. P. Barbalho, Philippi Sedir G. Morais, Gleyson J. P. Caldeira-Silva, Manoel H. Romão, Janaína L. R. S. Valentim, Aline P. Dias, Joaquim L. M. Alcoforado, Carlos A. P. Oliveira, Karilany D. Coutinho, Maria C. F. D. Rêgo, Ricardo A. M. Valentim

**Affiliations:** ^1^Laboratory of Technological Innovation in Health (LAIS), Federal University of Rio Grande do Norte (UFRN), Natal, Brazil; ^2^Department of Biomedical Engineering, Federal University of Pernambuco, Recife, Brazil; ^3^International Council for Open and Distance Education, Oslo, Norway; ^4^Centre for Interdisciplinary Studies, Faculty of Arts and Humanities, University of Coimbra, Coimbra, Portugal; ^5^Faculty of Psychology and Education, University of Coimbra, Coimbra, Portugal; ^6^Multi-Professional Institute for Human Development with Technologies, State University of Rio de Janeiro, Rio de Janeiro, Brazil; ^7^Graduate Program in Education of the Federal University of Rio Grande do Norte, Natal, Brazil

**Keywords:** health education, massive education, massive health education, massive online open courses (MOOC), syphilis, syphilis and other STI, learning path, syphilis epidemic in Brazil

## Abstract

With syphilis cases on the rise, Brazil declared an epidemic in 2016. To address the consequent public health crisis, the Ministry of Health laid out a rapid response plan, namely, the “Syphilis No!” Project (SNP), a national instrument to fight the disease which encompasses four dimensions: (a) management and governance, (b) surveillance, (c) comprehensive care, and (d) strengthening of educommunication. In the dimension of education, the SNP developed the learning pathway “Syphilis and other Sexually Transmitted Infections (STIs)” to strengthen and promote Health Education. This pathway features 54 Massive Open Online Courses (MOOCs), delivered through the Virtual Learning Environment of the Brazilian Health System (AVASUS). This paper analyzes the impacts of the learning pathway “Syphilis and other STIs” on the response to the epidemic in Brazil, highlighting the educational process of the learning pathway and its social implications from the perspective of the United Nations' 2030 Agenda and its Sustainable Development Goals. Three distinct databases were used to organize the educational data: the learning pathway “Syphilis and other STIs” from AVASUS, the National Registry of HealthCare Facilities from the Brazilian Ministry of Health (MoH), and the Brazilian Occupation Classification, from the Ministry of Labor. The analysis provides a comprehensive description of the 54 courses of the learning pathway, which has 177,732 enrollments and 93,617 participants from all Brazilian regions, especially the Southeast, which accounts for the highest number of enrollees. Additionally, it is worth noting that students living abroad also enrolled in the courses. Data characterization provided a demographic study focused on the course participants' profession and level of care practiced, revealing that the majority (85%) worked in primary and secondary healthcare. These practitioners are the target audience of the learning pathway and, accordingly, are part of the personnel directly engaged in healthcare services that fight the syphilis epidemic in Brazil.

## 1. Introduction

Brazil saw the rate of syphilis cases escalate alarmingly over the years 2011–2015, a phenomenon that led the country to declare a syphilis epidemic in 2016. Between 2010 and 2016 alone, the incidence rate of congenital syphilis (CS) and the case detection rate of maternal syphilis (MS) tripled, from 2.4 to 6.8, and from 3.5 to 12.4 cases per 1,000 live births, respectively. Case detection rates of acquired syphilis, notifiable since 2010, increased from 2.0 to 42.5 cases per 100,000 population (1, 2). Given this scenario, it is important to underscore that all types of syphilis are notifiable diseases in Brazil.

To address the ensuing public health crisis, the Brazilian Ministry of Health (MoH) laid out a rapid response plan, namely, the “Syphilis No!” Project (SNP), as a national instrument to fight the disease. The SNP encompasses four dimensions: (a) management and governance, (b) surveillance, (c) comprehensive care, and (d) strengthening of educommunication (3, 4).

In dimension (d), the SNP developed the learning pathway “Syphilis and other Sexually Transmitted Infections (STIs)” to strengthen and promote Health Education. This pathway features 54 Massive Open Online Courses (MOOCs), delivered through the Virtual Learning Environment of the Brazilian Health System (AVASUS). In view of educational needs, this learning pathway was designed to induce changes in work processes geared toward fighting syphilis. The strategy was to strengthen Continuing Health Education (CHE) to act as a tool to induce changes in professional practices (5). This process consisted of intervention activities aimed at promoting health policies related to the syphilis epidemic response in Brazil. In this vein, the elaboration of the SNP learning pathway considered expectations and educational needs regarding the quality of care to be delivered to the community. In that regard, it is worth noting the need for expanded testing to enhance the diagnosis of syphilis in Brazil, especially among pregnant women, as it is an essential factor in reducing the transmission rate of CS.

### 1.1. The “Syphilis No!” project

With cases of syphilis rising at a fast pace, the MoH developed a Rapid Response Project against the severe public health crisis. The project was approved in 2017 through a national and interfederative pact (3). In 2018, a Technical-Scientific Cooperation was established involving the MoH, the Federal University of Rio Grande do Norte (UFRN), through the Laboratory of Technological Innovation in Health (LAIS), and the Pan American Health Organization (PAHO) (4). One of the cooperation goals was to implement the “Syphilis No!” Project (SNP) as one of the national tools to combat syphilis with both local and universal actions. Local actions were carried out in the 100 municipalities considered a priority area by the MoH (municipalities that accounted for 68% of the cases of CS in the country) (2). Universal actions (nationwide) were implemented in all 5,570 municipalities of Brazil, 26 states, and the Federal District (6).

**Figure 1:**
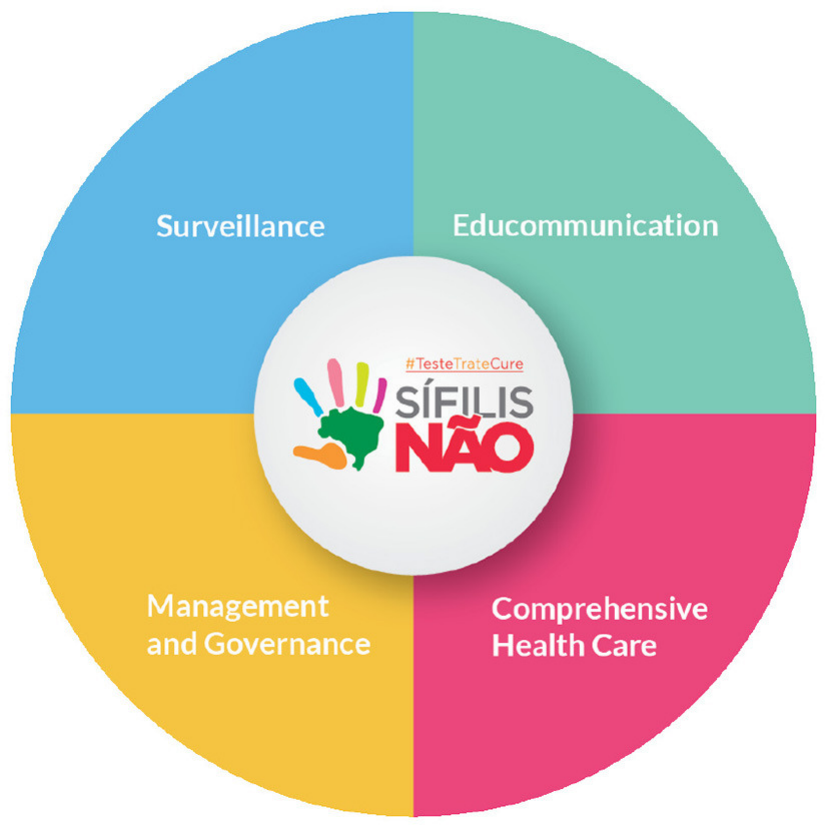
“Syphilis No!” Project development dimensions.

As illustrated in Figure 1, the SNP was developed, essentially, according to four dimensions: management and governance, surveillance, comprehensive care, and strengthening of education and communication (4, 7).

In the educommunication CHE dimension, the SNP developed activities toward training a qualified workforce across the Brazilian National Health System (SUS, by its Portuguese acronym) to face the syphilis epidemic. Implementing this dimension resulted in the elaboration of the learning pathway “Syphilis and other STIs,” consisting of 54 Massive Open Online Courses (MOOCs) (8). MOOCs include courses offered in virtual environments that enable the development of self-learning strategies. They, therefore, can be used for massive and free of charge education of health workers, particularly in health crisis settings (9–11).

The learning pathway on “Syphilis and other STIs” is available at https://avasus.ufrn.br/local/avasplugin/cursos/sifilis.php (12). The pathway was offered through the Virtual Learning Environment of the Brazilian National Health System (AVASUS, available at: https://avasus.ufrn.br). AVASUS is an official MoH platform that, in 2021, surpassed more than one million enrollments in more than 300 courses (13). Despite being a national platform, AVASUS has also reached students in countries around all five continents, as all AVASUS educational offerings are provided in an online format (8, 14).

This study is grounded on the following assumption: massive open online health education supported by AVASUS and its technological mediation, through the learning pathway “Syphilis and other STIs,” will positively affect the fight against the syphilis epidemic in Brazil. In this way, our main guiding question is: what are the impacts of the learning pathway “Syphilis and other STIs” on practitioners' work processes from a public health perspective?

### 1.2. Characterization of the learning pathway “Syphilis and other STIs”

The use of MOOCs is a reality, especially to meet the training and continuing education demands of health professionals in Brazil and worldwide (10, 11, 15). In their review of health-related MOOCs, Goldberg et al. (16) identified the importance of learning verification metrics and evaluative measures. Valentim et al. (17, 18) highlighted the need for improving scalability. Longhini et al. (19) mapped out the value of MOOCs in undergraduate and graduate health sciences programs, as well as for nurses and other health professionals' continuing education. Rossettini et al. (20) examined MOOCs in the context of physical therapy education and identified potential shortcomings yet to be overcome, such as social inequalities and participant evaluation.

The Learning and Capacity Development for Health Emergencies Unit at the World Health Organization has invested heavily in developing flexible MOOCs, in record time, to respond to the COVID-19 emergency (21, 22). In their recent scoping review of health-related MOOCs in low- and middle-income countries, Nieder et al. (23) documented their wide acceptance, especially when used to address public health crises, such as those caused by conditions like plague, diphtheria, ebola, and COVID-19. Findings such as these supported our development of a series of MOOCs to provide a learning pathway to address the public health crisis of the syphilis epidemic in Brazil.

A learning pathway is a systematic set of educational resources in which the student has the opportunity and autonomy to move through learning modules according to their goals and objectives (24–27). Nabizadeh et al. (28) describe it as the implementation of a curricular design that consists of a set of learning activities that assists course participants in achieving specific learning objectives with a solid educational and formative intentionality.

In the context of the AVASUS, the “Syphilis and other STIs” pathway was part of structuring the teaching-learning process, one of the universal interventions to spur health policy to qualify the SUS workforce in response to the epidemic (8). This learning pathway consists of a highly diverse and dense pedagogical architecture covering different thematic dimensions related to syphilis and other STIs. Further information about the courses analyzed can be found in the supplementary file “Learning Pathway Syphilis and other STIs.”

As the courses offered in this learning pathway and all other AVASUS courses are open and freely available, anyone interested in the topic can enroll in a variety of courses. Its pedagogical architecture was designed to essentially include interventions to promote the national policy to fight syphilis in the educommunication dimension (8). In this context, the educational offerings foreseen in such a pedagogical architecture encompassed these central topics:

- Key Populations and Vulnerable Populations: National Policy on Comprehensive LGBT Health; Men Who Have Sex with Men (MSM), and the Prison System;
- Syphilis in Pregnancy and Prenatal Care;
- Mother-to-child Transmission of Syphilis;
- Congenital Syphilis;
- Tests and Diagnosis;
- Public Health Surveillance and Primary HealthCare; and
- General Information on Syphilis and other STIs.

Of the 54 available courses launched on May 12, 2016, and available on April 25, 2022, 51 were included in this study. The remaining three were released after data collection. The availability of new courses in the learning pathway being analyzed follows a constant flow according to the needs that the coordination of the SNP or the students identify in their course evaluations on AVASUS.

Combined, the courses offered in the learning pathway account for 1,220 h of workload, grouped as follows (available at: https://avasus.ufrn.br/local/avasplugin/cursos/sifilis.php):

1. Two courses (3.92%) consist of over 60 h;
2. Seven courses (13.72%) range from 30 to 60 h;
3. Eleven (21.56%), from 15 to 30 h; and
4. Thirty-one (60.78%) have <15 h course loads.

Given the range of educational offerings, the need to analyze the impact of the CHE promoted by the learning pathway on the health service emerged. In order to assess the effectiveness of health education, one must understand that the education-health dyad forms an epistemic field of significant relevance to public health policy-making (29–31). Hence, studying these dimensions, in the context of the SUS workforce, along with the effects they have on health services and public health, represents an indispensable step in assessing the resilience of work processes (practice) and, therefore, of the health system itself (32–34).

From the perspective of (35, 36), the impacts of CHE must be measured beyond the outputs in terms of the learners' performance in the courses and the consequent changes in work processes in their practice settings. Analysis and understanding of the context are needed. That means taking into account epidemiologic and public policy aspects related to the program under study, by reflecting and seeking out answers on how and why such transformations have been occurring in parallel with educational activities.

In this context, the paper presents an impact evaluation of the learning pathway “Syphilis and other STIs” from the perspective of the United Nations' (UN) 2030 Agenda and its Sustainable Development Goals (SDGs), based on two perspectives:

1. Results of the courses offered: the reach of MOOCs in terms of education of health professionals; the spatial distribution of enrollees across Brazil; the levels of care covered (Primary, Secondary, and Tertiary HealthCare);
2. Discussion and context analysis: the educational process of the learning pathway and its relationships to the syphilis epidemic in Brazil, particularly the elimination of mother-to-child transmission (MTCT) of syphilis.

## 2. Materials and methods

This section describes data processing procedures according to data sources and the computational and statistical methods used for data analysis.

**Figure 2:**
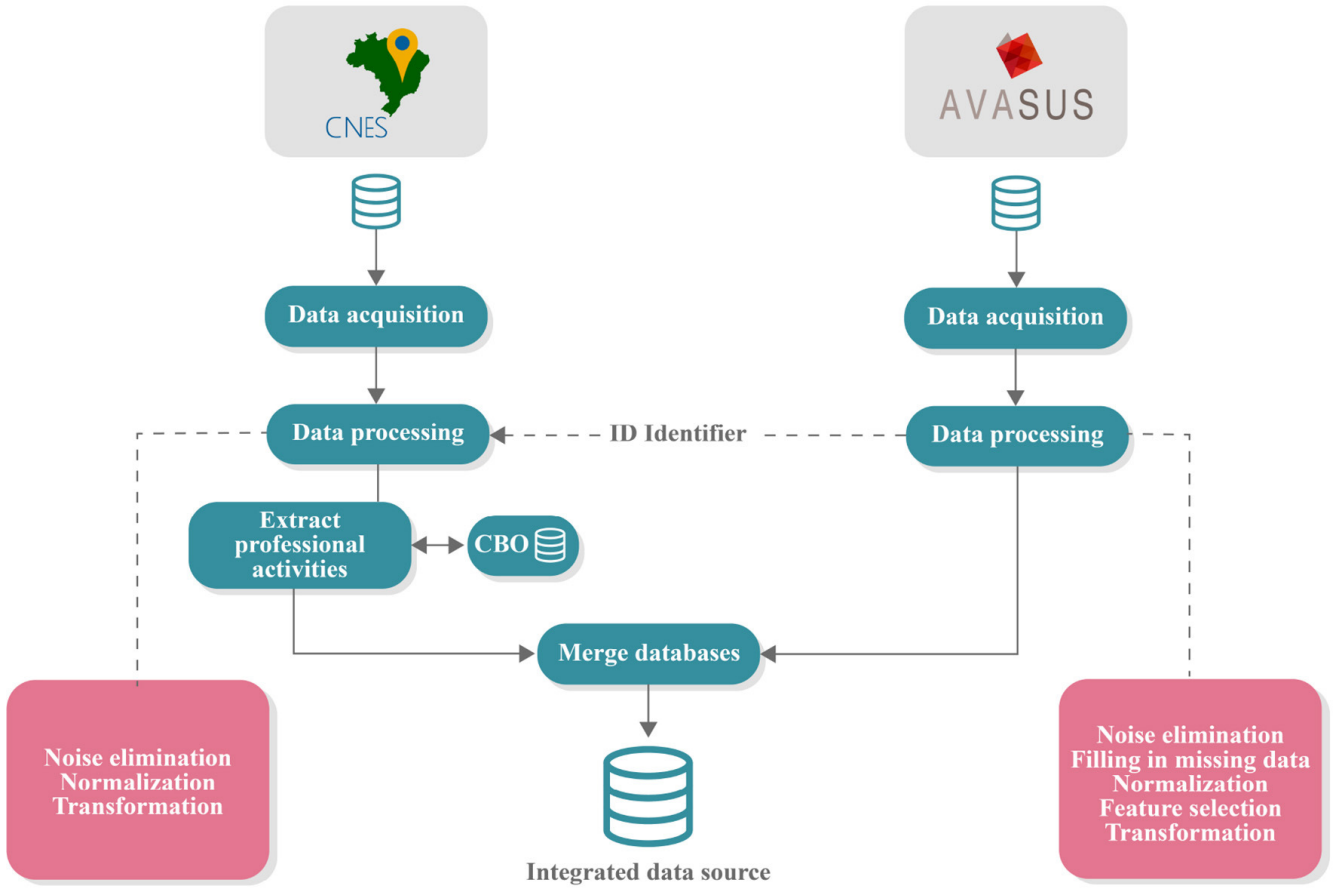
Data acquisition and processing pipeline. CNES, national registry of healthcare facilities; AVASUS, virtual learning environment of the Brazilian health system; CBO, Brazilian classification of occupations.

For a thorough and robust data analysis, it was necessary to profile the course participants registered in the learning pathway. We considered their field of practice and the levels of care delivered in their health settings (primary, secondary, and tertiary care). Figure 2 shows a flow chart illustrating methods for processing and structuring educational data.

Three different databases were used in structuring the educational data:

1. Data from the learning pathway “Syphilis and other STIs” from AVASUS;
2. Data from the National Registry of HealthCare Facilities (CNES) (37) of the MoH, and
3. Data from the Brazilian Occupation Classification (CBO) (38) from Brazil's Ministry of Labor (MoL).

Before being collected, all data from AVASUS were anonymized, and use was authorized by the coordination of the SPN. The CNES data are in the public domain, made available by the Brazilian MoH. The CBO data are also publicly available by the MoL. After data processing, the compilation from the three sources resulted in a single database with all the structured data, which was then published in a public domain repository (Zenodo) for use in further studies on this topic. It is available at: https://doi.org/10.5281/zenodo.6549079.

In order to interrelate the professionals in both databases, a unique identifier (ID) was assigned to each course participant when AVASUS exported data. This ID allowed each participant to be identified in the CNES and the CBO database. The CNES database stored the occupation codes of each practitioner, the health facilities in which they operated, and the category of health facilities according to the services provided. The CBO codes recorded in CNES enabled the identification of the occupation descriptions in the CBO database (see Figure 2).

The present study elicited syphilis testing data in addition to epidemiological data on this disease. The investigation of this information has proven fundamental in the impact analysis of CHE for professionals since it represents a relevant piece of evidence of changes in work processes and, thus, in the fight against the epidemic around Brazil.

### 2.1. Characterization of educational data

The educational data were extracted from AVASUS (8) and covered the period from May 12, 2016, to January 14, 2022 (although only data through 2021 were analyzed). At first, this dataset was composed of 103 attributes (features) and 177,732 instances (occurrences). The attributes corresponded to the information of the courses in “Syphilis and other STIs” course participants and respective enrollments. The instances corresponded to the enrollments existing in the database. Figure 2 breaks down the pipeline of essential steps for data analysis.

Initially, a data processing step was performed to eliminate noise, identify missing data, standardize the data, and select the essential attributes for data analysis. At the end of this step, we realized it was necessary to seek supplementary sources to complement the data and provide robustness to the study. The supplemental data (e.g., CBO) were extracted from the CNES database. After retrieving the information from CNES, the dataset underwent a processing step. This step used the ID of participants in the learning pathway courses to retrieve data from the CNES database and then obtain information about the health facility code and the numerical key relating to the profession of the course participants from the SUS workforce.

To identify participants' occupation, it was necessary to stratify the active professionals by consulting the CBO database. Lastly, the supplementary data from the CNES were integrated into the AVASUS database through the “ID” of the course participant to support data analysis. After this step, the resulting database contained 16 attributes (features) and 177,732 instances (occurrences), available at: https://doi.org/10.5281/zenodo.6549079.

#### 2.1.1. Incidence: Rate of course participants in relation to population size

To verify course participation, due to the population variation between Brazilian regions, it was necessary to determine the appropriate proportion of enrollment data in relation to the population of its respective area. This process prevented biased information from being generated. For instance, regions with larger population size and a larger health workforce tend to have a more significant number of enrollees, yet this does not mean more trained health professionals per 100,000 population.

From that perspective, it was necessary to normalize enrollment data per 100,000 population in relation to the country and each region. And also, for comparison purposes, we calculated the incidence based on the number of enrollees in the courses in the pathway, according to the notes in Equation (1). This incidence measures the rate of course participants in relation to the area's population size (in this instance, Brazil or the Brazilian region) per 100,000 population.


(1) $$\begin{array}{l} incidence=\left(\frac{x_{enrollment}}{x_{pop}}\right)n_{factor} \end{array}$$

were:

– *incidence*: variable used to store the coefficient related to the proportional indicators for each region or Brazil;
– *x*_*enrollment*_: variable used to determine the value corresponding to the number of enrollments;
– *x*_*pop*_: variable used to determine the value for each region's population;
– *n*_*factor*_: variable used to determine the factor of proportionality.

### 2.2. Characterization of epidemiological data

The epidemiological data on syphilis case notifications used for this study were collected through the Notifiable Diseases Information System (SINAN) from the MoH (39, 40).

For this study, we considered the case reports of MS and CS in the period from 2016 to 2020 with monthly time aggregation. The lower limit of this temporal cut-off was the year 2016, according to the onset of content production in AVASUS focused on syphilis and other sexually transmitted infections (STIs). The upper limit consists of 2020 due to the consolidation of epidemiological data on syphilis. In Brazil, the MoH consolidates these data in the subsequent year; for example, the 2020 data were consolidated in the second semester of 2021.

### 2.3. Characterization of syphilis testing data

Syphilis testing data were elicited from the SUS outpatient data (41). National records for the period from January 2016 to October 2021 were considered according to the methods used for syphilis detection in Brazil, which are:

- Fluorescent Treponemal Antibody Absorption (FTA-ABS) IgG
- FTA-ABS IgM
- Non-treponemal tests
- Rapid Point-of-Care testing
- Non-treponemal tests

The sum of the number of all types of tests performed was grouped by month so as to allow its analysis alongside the other mentioned data sources.

### 2.4. Statistical analysis

The Shapiro-Wilk test was applied to the number of enrollments in the learning pathway courses and the ratio between CS and MS to check for normal distribution (42). Both samples tested showed non-normal distribution, requiring the use of a statistical test that does not assume a normal distribution of data. In this case, we adopted Spearman's test (43) since it is a widely used correlation test that met the experiment's needs. For validation of all tests used in this study, a *p* < 0.05 was considered statistically significant.

## 3. Results

### 3.1. Reach, spatial distribution, occupation, and level of care

**Figure 3:**
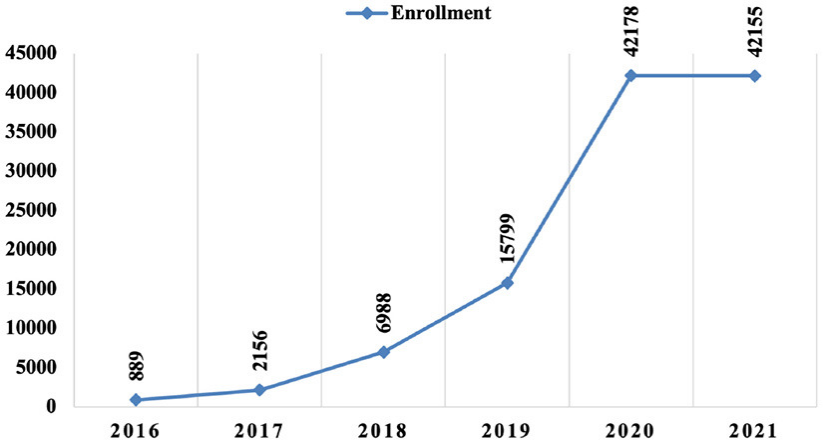
Trends in enrollment in courses in the learning pathway “Syphilis and other STIs” (2016–2021).

After data analysis and processing, it was possible to characterize the 51 courses that were part of the AVASUS learning pathway “Syphilis and other STIs.” According to the data, from May 12, 2016, to January 14, 2021, enrollees who had completed at least one course on the platform totaled 93,617 people. Of note, 177,732 people enrolled in the courses available in the pathway. On average, course participants enrolled 1–2 of the 51 MOOCs. Figure 3 breaks down the increase in the number of enrollments in the learning pathway courses up to the last day of 2021. The year 2022 was not considered as enrollments corresponded to only 14 days of this same year in the interval under analysis.

All the participants in the learning pathway have spontaneously enrolled and participated in the courses. The entire process for participants' engagement was achieved through information dissemination on AVASUS, on social networks of the SNP, and also through the interfederative agenda agreed upon through the national policy to fight syphilis in Brazil (3, 6, 8).

The courses had students from all Brazilian regions and respective states. The Northeast region had the most significant number of participants, with 32,027 (34.21%), followed by the Southeast region, with 31,143 (33.27%), the South region, 13,416 (14.33%), the Midwest region, 9,234 (9.86%), and the North region, 7,482 (7.99%). People living abroad also enrolled in the pathway, accounting for 197 registrations (0.21%). The data analyzed also show that 118 (0.13%) of the participants did not identify their place of residence.

**Figure 4:**
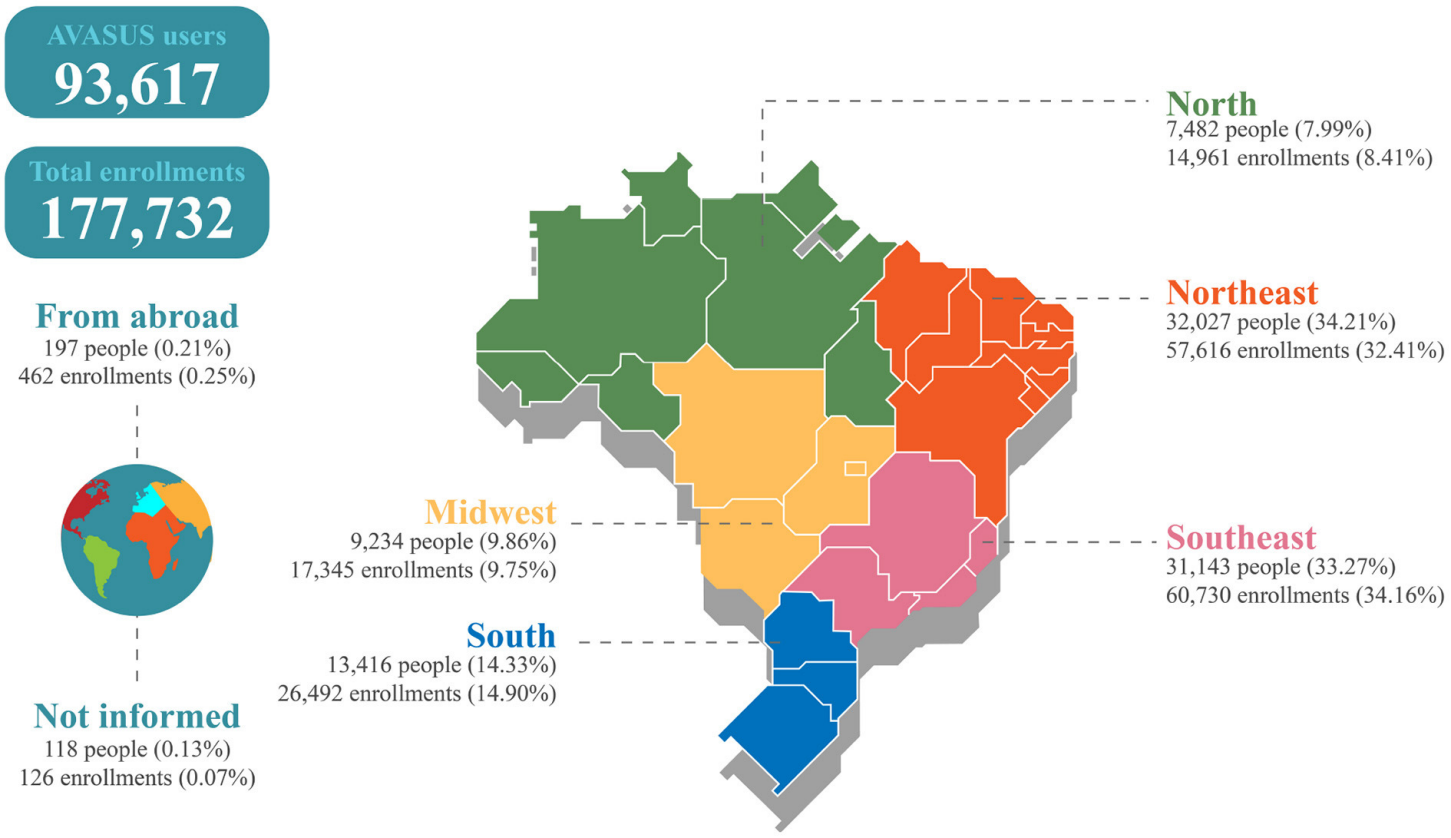
Geographical distribution of participants and enrollments in the learning pathway “Syphilis and other STIs.”

In terms of enrollment numbers, the Southeast figured the highest number of enrollments, with 60,730 (34.16%); followed by the Northeast, with 57,616 (32.41%); the South, with 26,492 (14.90%); the Midwest, with 17,345 (9.75%); and the North, with 14,961 (8.41%). Figure 4 details the spatial distribution of participants and their enrollments.

**Figure 5:**
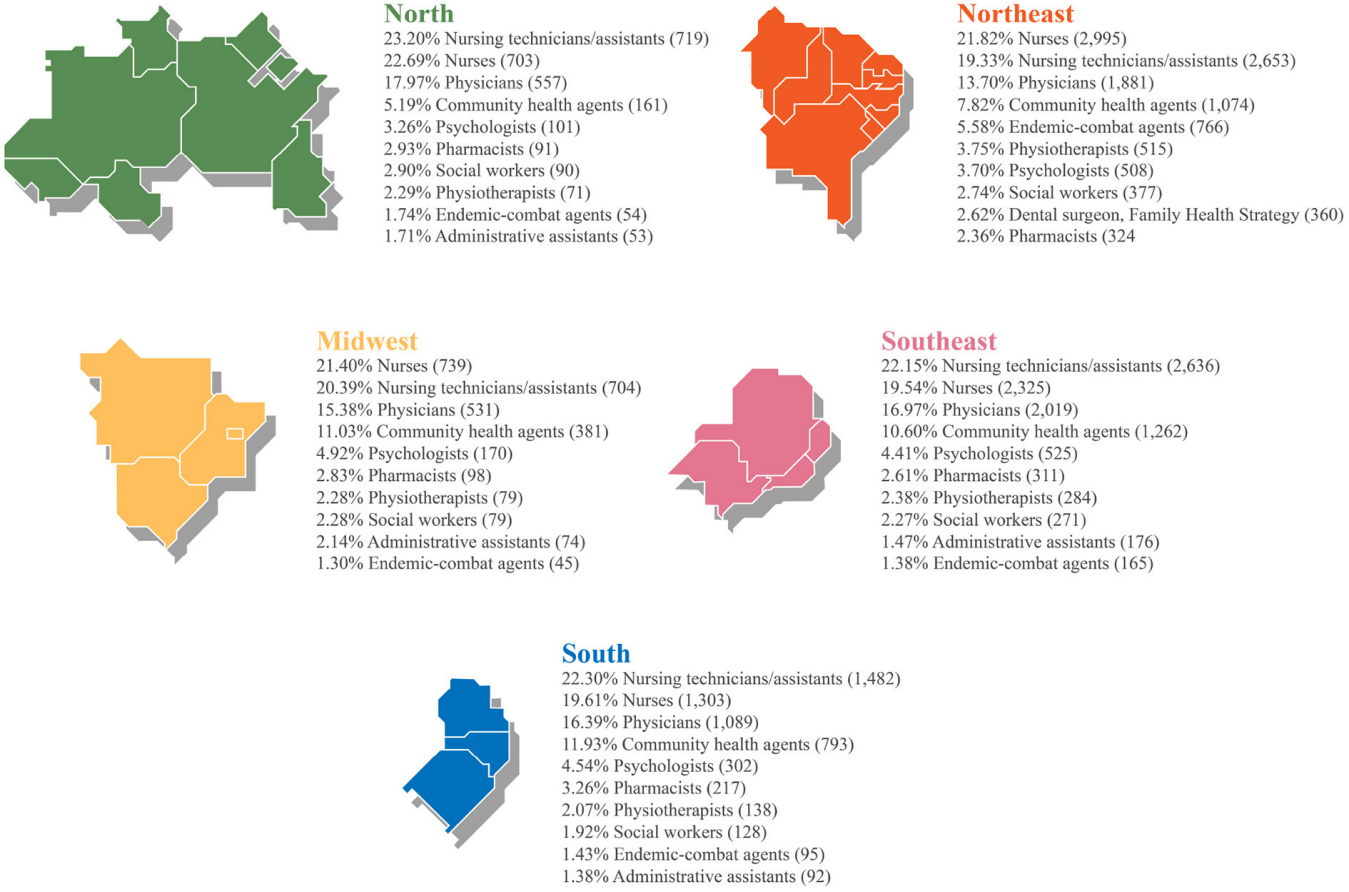
Ranking of participants' occupations by region.

**Figure 6:**
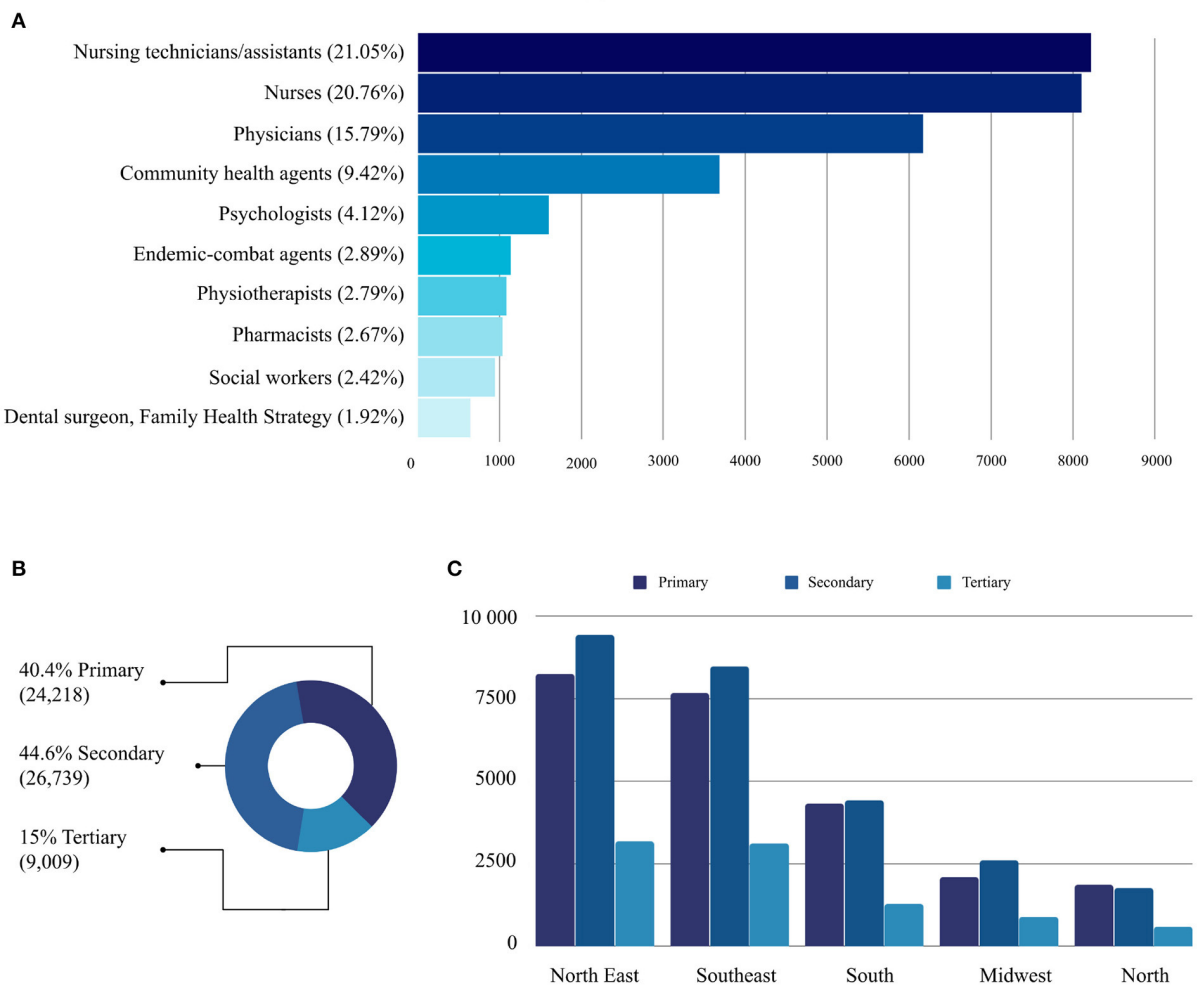
**(A)** Ranking of the ten most frequent occupations in the profile of participants in the pathway. **(B)** Level of care practiced by participants according to their health facility. **(C)** Geographical distribution by region of the levels of care practiced by course participants.

With respect to participants' occupations, we ranked the ten most common professions found in each Brazilian region (Figure 5) and in the country in general (Figure 6A). For this analysis, only the occupations identified were included. Thus, 54,641 (58.36%) professionals who filled “Not Informed” or “No Formal Employment” in the occupation field were not counted.

Figure 6A shows that of the 93,617 participants, 21.05% were nurse technicians, and 20.76% were nurses. This >41% outcome is particularly relevant because, in SUS, this workforce operates on the front line of healthcare networks combating syphilis in Brazil. When added to the number of physicians (15.79%), who are also professionals active in facing syphilis, the technological mediation provided by AVASUS has reached at least 55% of targeted practitioners (nursing technicians, nurses, and physicians). If Community Health Agents (CHAs) are included, this percentage goes up to 65%. Accordingly, a closer examination of the five regions shows that the four most frequent occupations at country level (general) are also those most frequent at regional level (specific), changing only the order (Figure 5).

In Brazil, CHAs work directly in the community; they are part of family health teams. Therefore, they represent the SUS workforce specifically operating in primary healthcare (44). CHAs are also responsible for actively searching for pregnant women who have initiated prenatal care and did not attend the health center on the scheduled appointments (45).

Another key aspect evident in Figure 6B is the level of healthcare in which participants were operating. According to the guidelines established by the MoH, in Brazil, health facilities are categorized by level of care, namely (46):

1. Primary Health Care (PHC): corresponds to the Family Health Centers (USF), Fluvial Health Centers, Mobile Dental Services, and the Health Fitness Centers;
2. Secondary Health Care (SHC): corresponds to the Emergency Care Units (UPA), hospitals, and other specialized care or medium complexity services, and
3. Tertiary Health Care (THC): includes larger hospitals or hospitals with high complexity services, which can be either publicly or privately subsidized.

The number of participants with occupations at the PHC and SHC was more pronounced. As shown in Figure 6B, the learning pathway courses attracted more participants with occupations in primary and secondary healthcare. Figure 6C highlights the geographic distribution, grouped by region and level of care, where participants operated. A similar trend can be observed across all regions. Tertiary care (high-complexity services) accounted for the lowest number of participants. This is mainly because the courses available in the learning pathway are not oriented to health professionals working in high-complexity services.

Primary and secondary care professionals are key in ensuring that pregnant women receive adequate and high-quality prenatal care, which is considered an essential element of the public health agenda to eliminate MTCT transmission of syphilis (47–50).

### 3.2. Educational processes

**Figure 7:**
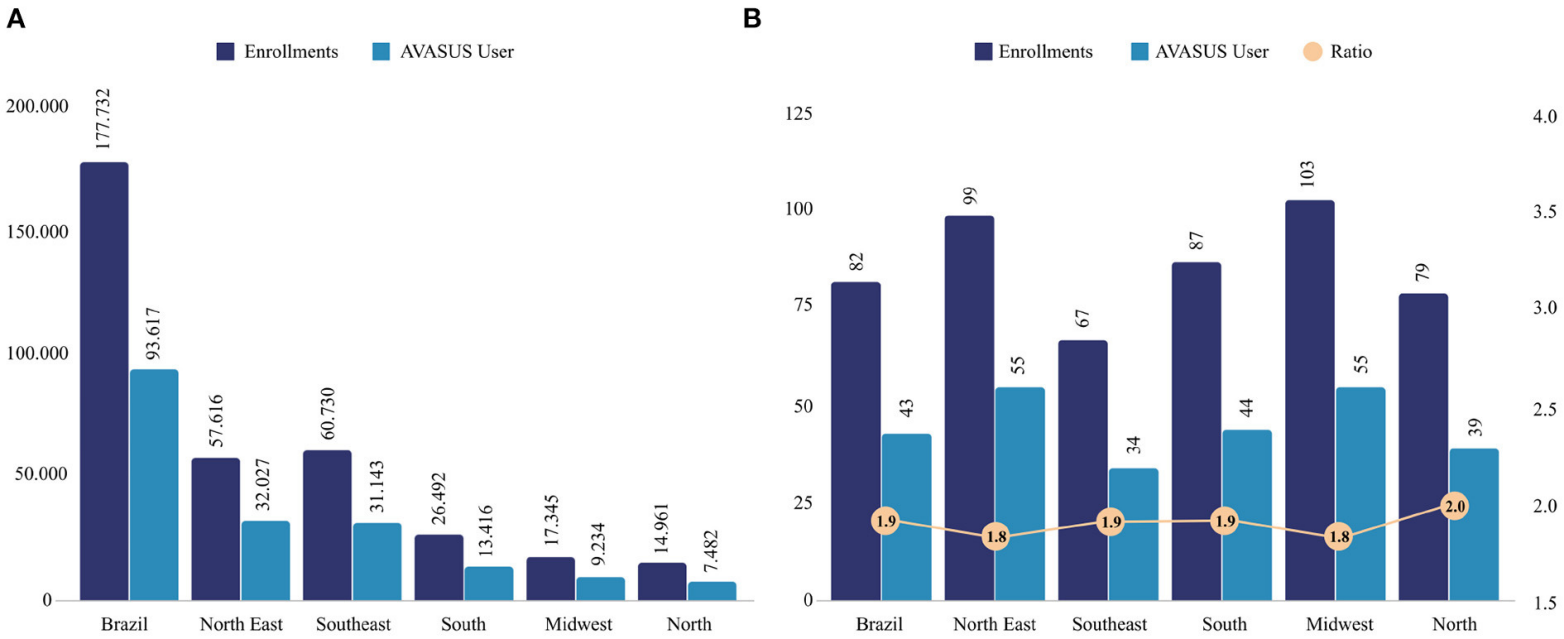
**(A)** Number of AVASUS enrollments and participants. **(B)** Incidence of enrollments and participants in the AVASUS learning pathway (per 100,000 population) and the ratio between enrollments and participants.

In comparison to the total population, the Midwest region had the highest incidence of enrollments (103), followed by the Northeast (99), South (87), North (79), and the Southeast (67). Figure 7A presents the absolute numbers of enrollments and participants in the “Syphilis and other STIs” learning pathway. Figure 7B provides the incidence (per 100,000 population) of registrations and participants in the same pathway and the ratio between enrollment and participants.

If one observes only the absolute values (Figure 7A), it seems that the Northeast would be the region with the highest participation numbers. However, by examining the incidence (Figure 7B), it can be seen that the average variation (1.88) among the regions follows the same trend as for the country. The North stands out with the highest incidence but not significantly higher than the average incidence in the country.

Table 1 describes the courses with the lowest enrollment. When observing the “Start Date” column, it is noticeable that these are courses whose launch date in AVASUS was close to the data collection date (<6 months apart). Enrollment in “Syphilis and other STI” courses is continuously open. Therefore, the number tends to increase depending on interest, dissemination, and the period elapsed from the offer.

**Table 1:** Courses with lowest enrollment numbers.

**Course**	**Number of**	**Start**
	**enrollments**	**date**
Let's talk about syphilis: behavior and education in syphilis prevention	570	11/12/2020
Protección social de personas en situación de vulnerabilidad social con sífilis, VIH/SIDA, hepatitis viral, tuberculosis o lepra (social protection for people with syphilis, HIV/AIDS, viral hepatitis, tuberculosis, or Hansen's disease in situations of social vulnerability)	484	08/27/2021
The detention officer and health in Correctional Facilities	469	11/25/2021
Course on comprehensive care for people with sexually transmitted infections	401	09/16/2021
Social protection for people with syphilis, HIV/AIDS, viral hepatitis, tuberculosis, or Hansen's disease in situations of social vulnerability	2	08/27/2021

Table 2 lists the ten courses with the most enrollments for the analyzed period. These courses represent nearly 60% of the total registrations (177,732) for the period considered. Compared to the courses shown in Table 1, it can be noted they are the first courses available in AVASUS. This factor explains the higher participation since enrollment is continuously open.

**Table 2:** Courses with highest participation numbers.

**Course**	**Number of**	**Start**
	**enrollments**	**date**
Care of people living with HIV/AIDS in primary healthcare	24,707	09/24/2018
National policy for comprehensive LGBT health	15,724	05/12/2016
Primary healthcare, family health strategy, and territorialization	11,760	14/03/2019
Expanded clinics and matrix support	11,240	08/14/2017
Prenatal and puerperium at times of the COVID-19 pandemic	9,077	07/08/2020
Healthcare of persons deprived of liberty	8,118	06/07/2018
Congenital syphilis: from prenatal to outpatient monitoring	7,709	11/17/2019
Pre-Exposure Prophylaxis (PrEP) for HIV infection risk: training for health professionals	6,389	06/27/2020
Course on comprehensive care for people living with sexually transmitted infections	6,233	07/20/2021
Syphilis: pathogenesis, immune development, and diagnostic methods	5,806	12/19/2019

Of note, the course “Prenatal and Puerperium at times of the COVID-19 pandemic” (Table 2) was launched during the pandemic, in effect, to reinforce care provision during this crucial period for women. There had been a great deal of concern in Brazil that the health system was more fragile for Prenatal and Puerperium care management issues. Hence, the objective was to train health professionals on this topic during the major global health crisis brought on by COVID-19.

### 3.3. Course evaluation by participants who completed

**Figure 8:**
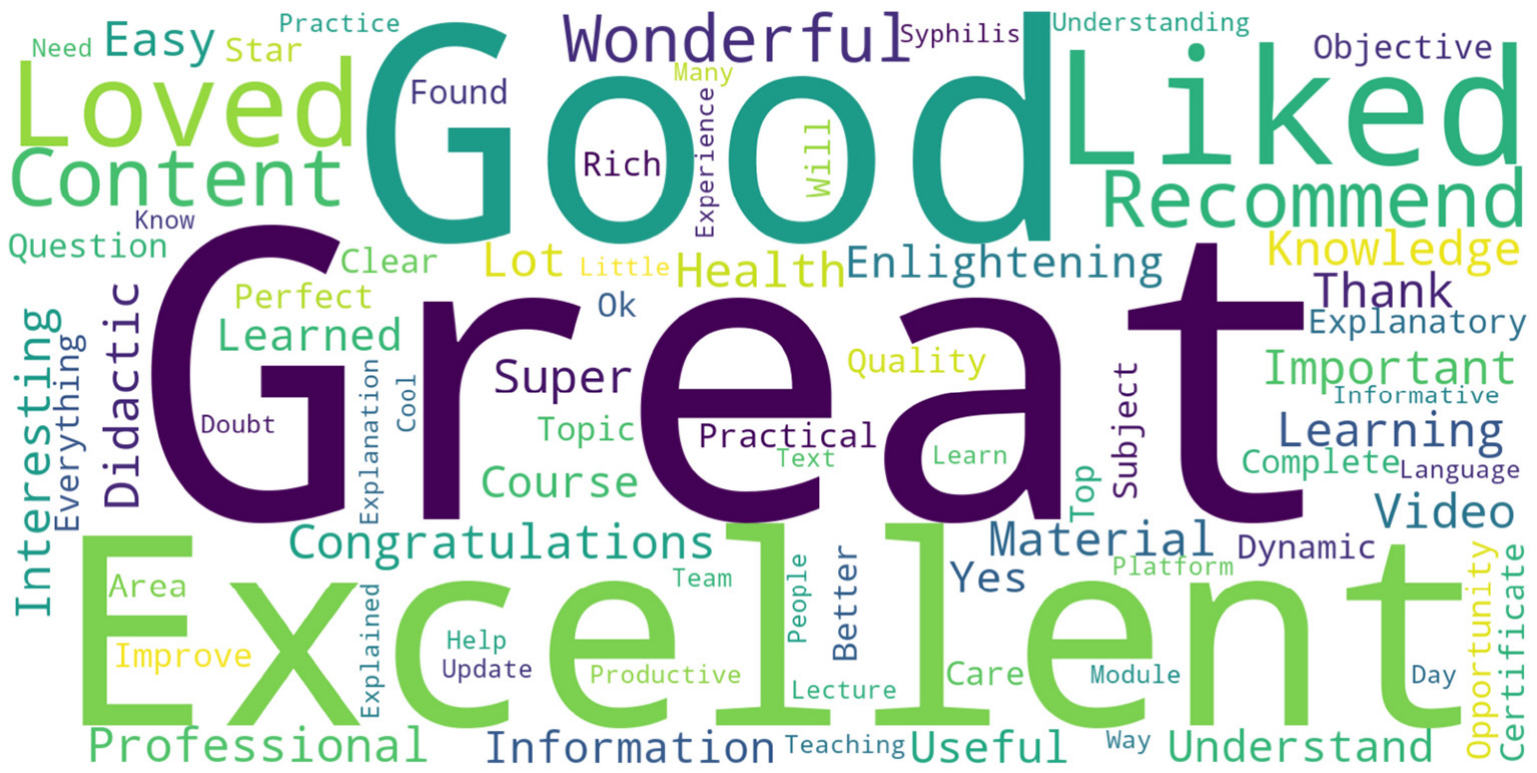
Word cloud of evaluative feedback from course participants.

A total of 177,732 people enrolled in courses on the pathway, and of those, 111,494 (62.73%) completed them. Of the completers, 99,698 (89.42%) provided evaluations, assigning a score from 0 to 5 (with zero being the lowest and five the highest). With courses collapsed, the average rating was 4.93, and the standard deviation 0.36. Students could also write a comment (free text) that represented their overall perception of the course when evaluating. Out of the 99,698 evaluators, 42,329 (42.46%) submitted their feedback. The word cloud shown in Figure 8 stems from these comments and highlights the participants' primary feelings toward the courses: great, good, excellent, content, loved, liked, recommend, and wonderful.

### 3.4. Analysis and impacts on public health

This section presents health surveillance data on syphilis in Brazil that help evidence changes within health professionals' work process. In turn, such changes may explain how massive open online health education has also contributed to decreasing congenital syphilis cases across the country.

**Figure 9:**
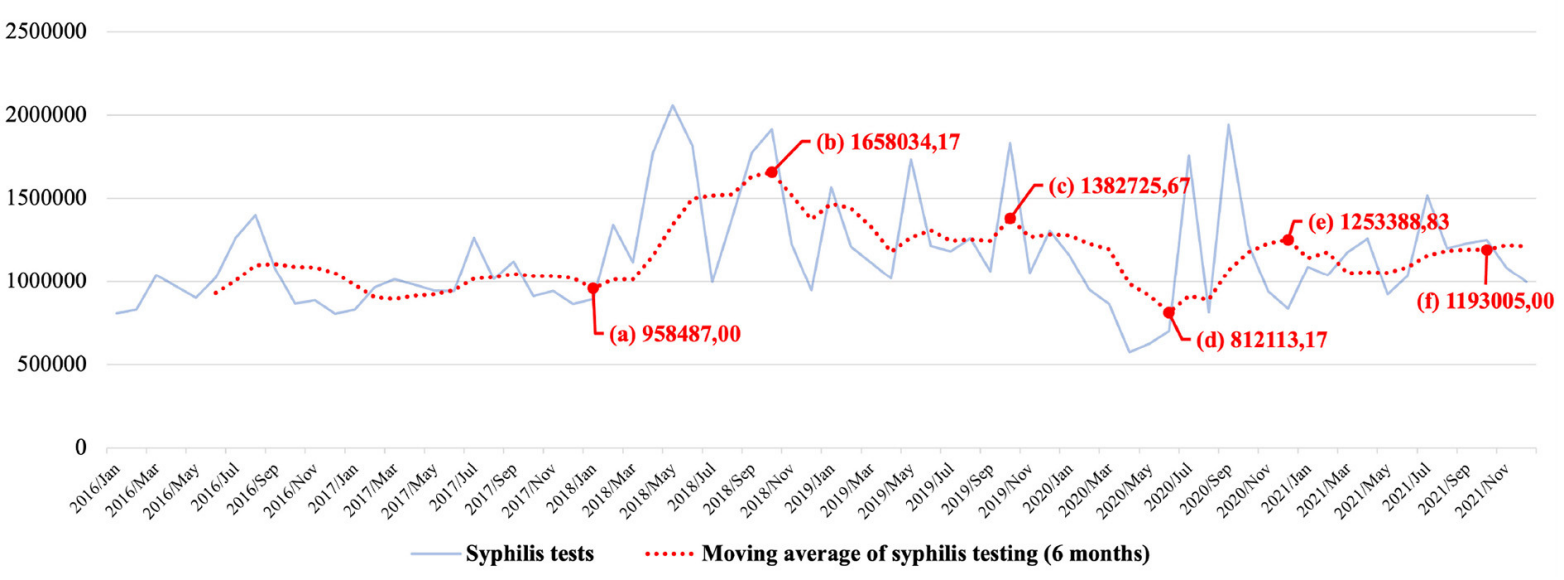
Tests for syphilis detection in Brazil.

In 2016, after the already declared Syphilis epidemic, there was a significant change in the increase in testing for each of the tests listed above. Figure 9 illustrates the growth in numbers related to testing. It can be seen that there is a highlighted point of inflection in the moving average of the graph (Figure 9a) that demonstrates a substantial change in trend in testing for 2018, coinciding SNP launch. This increase peaked at the end of 2018 as well, as shown in Figure 9b.

By the end of 2019, in a second peak, a major reduction in testing was recorded (see Figure 9c). Shortly after this peak, a drop in testing was recorded in 2020, between March and July (Figure 9d). This can be explained by the strong impact of the COVID-19 pandemic on health services, which needed to reorganize and restructure to absorb more of this demand. A fact that should be noted is that after the reduction in testing, in the first semester of 2020, an increase in testing for syphilis was registered again, which demonstrates the resilience and responsiveness of the Brazilian health system (33, 51), in the face of the severe global health crisis, but which has not failed to respond to the epidemic issue as well.

Recent studies by Pinto et al. (3) show that in 2015, the number of syphilis tests performed in Brazil was 518,859, which increased to 911,420 in 2016, and 1,448,364 in 2017. Such an increasing trend continued until 2019, with 2,533,571 tests. Aligned with this phenomenon, starting in 2018, the number of enrollments in “Syphilis and other STIs” courses began to grow considerably. Concerning syphilis testing in Brazil, this aspect was also observed in 2018 and 2019.

The fall in syphilis testing caused by the pandemic observed between January and June 2020 was overcome still in the second semester of the same year (see Figure 9e). Even with this reduction, 2020 still had more tests than 2016 and 2017 on average, with 4.37 and 5.03%, respectively, more tests than in those years before the pandemic.

Figure 9 shows a steady number of syphilis tests performed in Brazil. Despite showing a lower figure in 2021 compared to 2018 and 2019, the total number of tests is still above those of 2015, 2016, and 2017, by 7.87, 17.02, and 14.54%, respectively.

From 2015 to 2017, besides not being in the context of the pandemic, there was also no intervention by the SNP. It is essential to consider the COVID-19 pandemic because its effects could have dramatically affected the syphilis testing policy in the country; however, according to the data presented in Figure 9, this did not occur, at least not to a greater degree. According to data from the SUS outpatient production (37), shown in Figure 9, it can be seen that even during the COVID-19 pandemic, the SUS maintained a higher testing level than in the years when the SNP was not in operation.

The increase in testing and the reduction in CS case notifications, added to the massive increase in enrollment in courses in the learning pathway in 2019, strongly suggest beneficial changes in work processes in healthcare networks in Brazil. In particular, in PHC and specialty care (SHC), which are primarily responsible for delivering services such as syphilis testing, diagnostic, and treatment.

**Figure 10:**
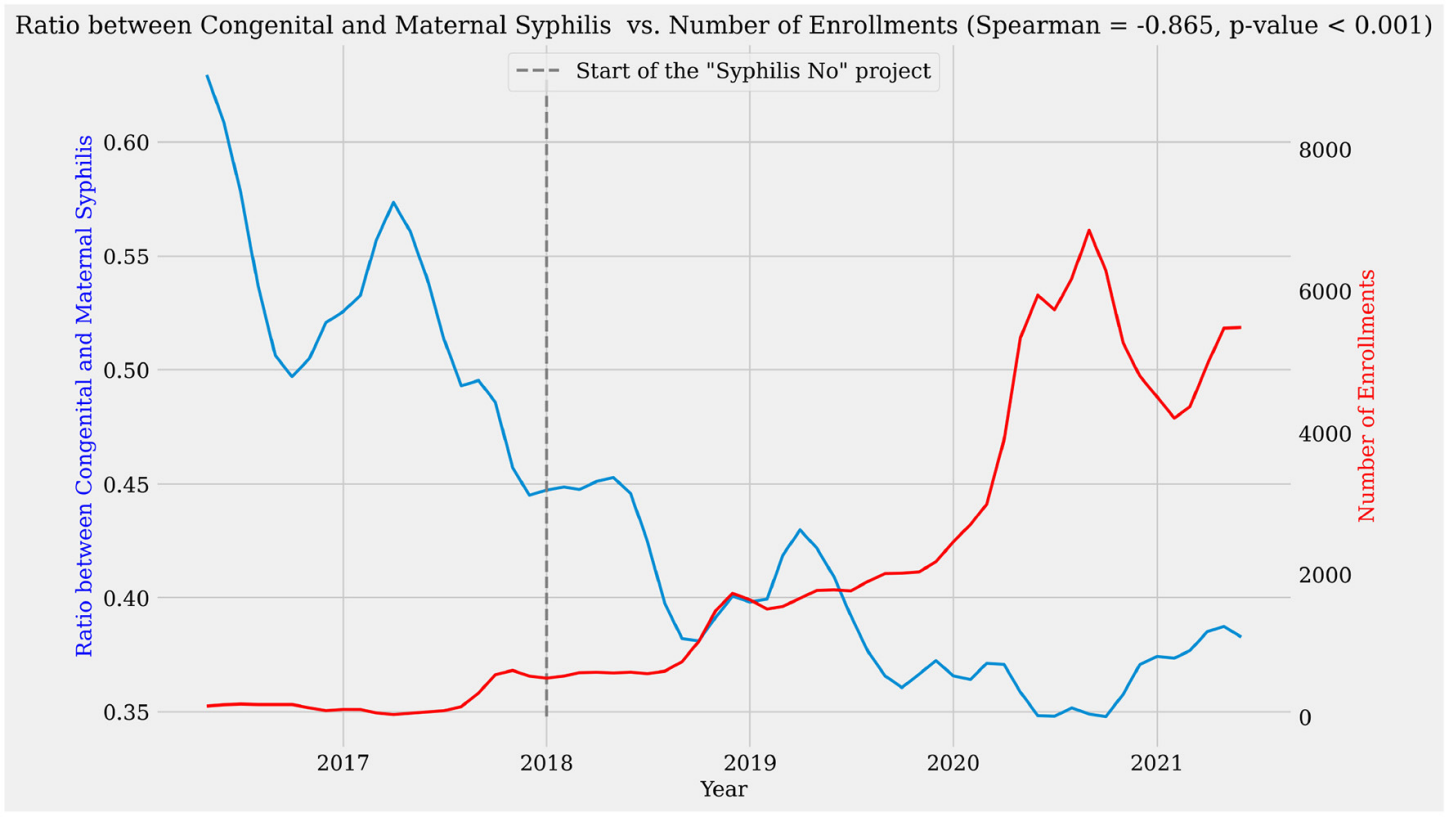
Correlation between the number of enrollments in the learning pathway and the ratio of CS and SIP case notifications.

Figure 10 evidences a negative Spearman's correlation of −0.865 between the number of enrollments in the courses in the learning pathway and the ratio between CS and MS evaluated in the period from May 2016 to June 2021. Thus, a strong correlation (52) with statistical significance >99.9%. Note that both variables were tested for normality, with tests indicating non-normal distributions. Therefore, the values for the number of enrollments in the learning pathway courses (Shapiro-Wilk coefficient = 0.83, *p* < 0.00001) and the ratio between CS and MS (Shapiro-Wilk coefficient = 0.907, *p* < 0.00019) falls within the assumptions for the Spearman's rank correlation.

Although this high negative correlation does not demonstrate causality, it reinforces that positive impacts have been produced in care and assistance services delivered to pregnant people. Moreover, this occurred parallel to the increase in massive health education interventions mediated by AVASUS through “Syphilis and other STIs.” Therefore, it was found that, as the number of enrollments increased, starting in 2018, the MTCT of syphilis started to drop (see Figure 10). This positive impact on MTCT rates indicates resilience and responsiveness (51) due to changes in the SUS work processes.

## 4. Discussion

Based on all data presented in the results, it can be said these courses had massive participation nationwide. There was a uniform and consistent regional distribution when comparing the enrollment incidence, as shown by Figures 3–7. Figure 3 evidences that the COVID-19 pandemic was the period with the highest percentage increase in enrollments. In 2020 and 2021, for example, there was 366% more enrollment than in 2019. Accordingly, the pandemic had no impact on massive enrollment in the learning pathway courses.

To interpret the results relative to the process of massive open online education undertaken to confront the syphilis epidemic in Brazil, it is necessary to examine the context in which the 51 MOOCs occurred to comprehend the impact of the learning pathway “Syphilis and other STIs.” This discussion addresses two dimensions: the impact on public health in response to the epidemic, and aspects related to the impact of the MOOCs in Brazil in view of the Sustainable Development Goals included in the UN's 2030 Agenda.

### 4.1. Response to the syphilis epidemic in Brazil: Analyzes and impacts

In parallel with syphilis testing growing trends in Brazil, AVASUS recorded a strong upward trend in enrollments in the “Syphilis and other STIs” courses, especially in 2020 and 2021. Enrollment increases started in 2018 but became more robust in 2020. This phenomenon provides strong evidence that work processes are changing across Brazil, which helps to explain the consistent and increasing trend in syphilis testing, even during the COVID-19 pandemic. Therefore, the combined analysis of such data underpins the importance of massive health education as a strategic tool for promoting public policies in response to public health emergencies, such as the syphilis epidemic in Brazil.

Another interesting finding is that the courses related to key and vulnerable populations recorded the highest participation, namely, the “National Policy for Comprehensive LGBTQIA+ Health” and “HealthCare of Persons Deprived of Liberty.” This demonstrates that the strategy of offering massive technology-mediated courses on health—adopted to fight the syphilis epidemic—such as the ones mentioned has achieved a satisfactory level of participation.

Courses with central topics related to the syphilis epidemic response deserve special attention: they were among those with the highest participation and general information about syphilis diagnosis and its management in specialty and shared care. The increased demand for courses related to CS, prenatal, and pregnant people, and PHC is indicative of the fact that there was a genuine demand. Consequently, that readily explains the participation numbers and reinforces the importance of massive health education as a tool for promotion and intervention in public health policy. Actions of utmost relevance are essential in situations of sanitary crisis, just as the syphilis epidemic mentioned.

Regarding the syphilis epidemic response in Brazil, the use of the self-learning model supported by technological mediation—linked to massive health education and through a learning pathway specific to this topic—reveals not only to be timely but also to promote health system resilience (33, 51, 53). This observation is especially valid when we observe the increase in enrollment in AVASUS courses on syphilis, even during the pandemic. This occurred not only because of the availability of courses but also because of the process of intervention and promotion of national policy in response to the syphilis epidemic, which occurred even during the COVID-19 pandemic (6).

Several interventions of interfederative nature, coordinated by Brazil's MoH, occurred through the SNP during the pandemic. Rocha et al. (4) describe these public health intervention actions in the 100 priority municipalities to confront syphilis in Brazil. Among the activities these authors mention, the following stand out: the issues related to prenatal care, the elimination of MTCT, PHC, and the addition of the problem of syphilis to the public health agenda. Besides these local interventions, Pinto et al. (6) describe a set of universal interventions: communication and educational interventions and interfederative agreements and articulations between the Union, states, and municipalities (7). The latter aimed to include the response to the syphilis epidemic in the public health agendas of states and municipalities in Brazil.

The intervention agendas Rocha et al. (4) and Pinto et al. (6) reported also occurred during the COVID-19 pandemic and coincided with the themes approached on “Syphilis and other STIs.” This helps explain the increased enrollment in the earning pathway during the highlighted period, where health professionals' focus was directed toward the COVID-19 pandemic. In this context, it can be seen that the interventions of the Brazilian agenda for syphilis response may have acted to mitigate the impacts of the pandemic. This helps explain the increase in enrollment in the courses in the pathway alongside the rise in testing in the country.

Before the pandemic, especially in 2018 and 2019, Brazil had already recorded a significant increase in syphilis testing. Pinto et al. (3) found an overall 289.09% increase in numbers in 2018 and a 375.18% increase in 2019, both about 2015. By this, the authors indicate that, in 2018, 3.89 times more individuals were tested in Brazil compared to 2015, and that in 2019, 4.75 times more people were tested than in 2015. Such years stand out because they fall within the period the SNP was operating.

Still based on the epidemiological data on syphilis, and according to the studies by Pinto et al. (3) and Andrade et al. (1), in 2018 and 2019, the ratio between SIP and CS fell from 41.9% in 2018 to 39.4% in 2019. In 2019, this drop occurred even when the rise in the number of tests performed was considered. It implies that, in 2019, the decline in notifications for CS did not happen because of the reduction in testing. The drop in CS cases across Brazil in 2019 was also confirmed by data from the *Syphilis Epidemiological Bulletin* published by the MoH in 2021 (54). In a study based on a time series analysis, Pinto et al. (6) demonstrate a change in trends in CS case notification in Brazil in 2019, which is pertinent as this had not occurred in the country for years (since 2010).

In 2020, according to the Syphilis Epidemiological Bulletin (2021) (54), the detection rate of MS and incidence of CS (per 1,000 live births) were 21.6 and 7.7, respectively. Therefore, in 2020, the MS detection rate was slightly higher than in 2018 (*n* = 21.5). However, 2020 scored a lower CS incidence when compared with 2018 (*n* = 9.0) and 2019 (8.5).

The increase in MS detection rate and reduction in CS incidence in 2020 are relevant indicators for our analysis. This data suggests that the quality of treatment of maternal syphilis in PHC and the specialty care network has been improved. These levels of care accounted for ~84% of participants in the available learning pathway courses. Considering these data, it is possible to see that technological mediation, applied in massive health education of practitioners, has positively impacted the response to the sanitary crisis provoked by the syphilis epidemic.

The massive education promoted by “Syphilis and other STIs” was a strategic tool for stimulating public health policy in response to the epidemic. The massive training process contributed to a more resilient and responsive health system during such a health crisis. The scalability of the learning pathway, notwithstanding the ease of access to continuing education and the broad national scope, might not have performed likewise if only conventional educational methods had been used in Brazil.

### 4.2. Social impacts: A look at the Sustainable Development Goals (SDGs)

To analyze the social impacts from the perspective of the SDGs (55–57) means to reflect on how massive open online education (58, 59) has functioned and intervened in a transdisciplinary and transversal fashion in several societal dimensions. Therefore, it must transcend the quantitative analyses that MOOCs provide to delve into qualitative issues (transversal and transdisciplinary) and how education can influence societies. Thus, this section's analytical approach focuses on how the learning pathway “Syphilis and other STIs” has contributed to achieving the SDGs, a global commitment arising from the Millennium Development Goals since 2000.

To look at the 17 SDGs and their 169 goals is to comprehend the complexity and challenges proposed in the global call for all people to have quality of life. “Syphilis and other STIs” contribute directly to SDG 3—Good Health and WellBeing and SDG 4—Quality Education. However, the social impacts of the MOOCs also extend to at least four other SDGs, namely: SDG 5—Gender Equality; SDG 10—Reduced Inequalities; SDG 11—Sustainable Cities and Communities and SDG 17—Partnerships for the goals.

The public health intervention led by the “Syphilis No!” Project through MOOCs, offered through technological mediation (AVASUS), was articulated based on content and knowledge. Hence, qualifying hundreds of thousands of people, health practitioners, and the general population during a syphilis epidemic in Brazil meant boosting health system resilience and responsiveness, in the case of Brazil, the SUS.

Promoting health and wellbeing (SDG 3) through a public health intervention to respond to a syphilis epidemic using massive education (SDG 4) in a continent-sized country like Brazil means providing opportunities for equity, reducing inequalities, and promoting social justice. Likewise, it worked as a tool for national and international articulation to construct multilateral and interfederative technical cooperation (SDG 17). This aspect is necessary for the dissemination, promotion, engagement, and PAHO on of the topic of syphilis in state and municipal health agendas throughout the country. As Pinto et al. (6) and Rocha et al. (4) described, the SNP actions covered all 26 Brazilian states, the Federal District, and 5,568 municipalities between 2018 and 2020.

Adding syphilis in the public health agenda in a country like Brazil, with a large territory, population size, and regional and cultural diversities, is a complex task. Therefore, the strategy of massive health education favors a faster response to public health problems when thought of within a cooperation and partnership model. SDG 17 stands out when compared to the effectiveness of the 51 analyzed courses in the learning pathway. That is because most offerings were designed and produced through partnerships and national and international cooperation, as shown in the AVASUS credits. The possibility of working in technical cooperation encourages the participation of new knowledge and enhances the power of networking. Goals 17.6, 17.8, 17.9, 17.16, 17.17, and 17.18 (55) specifically address multisectoral partnerships, which occur through technology and professional training. Therefore, we denote an effective contribution and impact of the learning pathway “Syphilis and other STIs” in implementing these goals of SDG 17.

Furthermore, including the theme of syphilis in the national health agenda, with a comprehensive spectrum of human health education mediated by technology, contributes to ensuring a healthy life and promoting wellbeing for all at all ages. For this reason, SDG 3 (Good Health and WellBeing), encompassing several knowledge domains and society sectors, underscores the most significant challenges concerning humankind's global health and wellbeing. In this context, the learning pathway worked directly on prenatal and postnatal care and sexually transmitted diseases, the most important themes of this SDG. Therefore, direct relationships were established between the courses offered in AVASUS and the goals of this SDG. We highlight goals 3.1, 3.2, 3.3, 3.7, 3.c, and 3.d (55), which have a social impact on the following aspects:

1. reducing the overall maternal mortality ratio;
2. reducing avoidable deaths of newborns and children under 5 years of age;
3. fighting sexually transmitted diseases;
4. information and education, and the integration of reproductive health into national strategies and programs; and
5. health worker development, training, and capacity building in developing countries.

When properly planned and structured, massive open online health education contributes to ensuring access to inclusive, quality, and equitable education and promotes lifelong learning opportunities for all. Regarding this domain, SDG 4 understands education as permanent and of quality for all people. Therefore, using massive open online education as one of the leading and transversal dimensions in several public health agendas has been established as a strategy within the national syphilis response policy (8). Concomitantly, this SDG 4-related strategy contributed to ensuring equal access for all men and women to quality education in response to the syphilis epidemic. Thus, eliminating gender disparities in education and ensuring equal access to all levels of education contribute to the implementation of SDG targets 4.3, 4.4, and 4.5 (55).

Regarding massive open online education in health, the open educational resources (OER) used in “Syphilis and other STIs” contribute to and socially impact the implementation of SDG 5, which targets gender equality and the empowerment of all women and girls. This can be observed when one analyzes goals 5.6, 5.b, and 5.c of this SDG (55), which list challenges, essentially, to ensure universal access to sexual and reproductive health and to advance the use of core technologies to promote women's empowerment.

In a landscape of public health crisis, producing social impacts through health education necessarily implies facing and looking at social inequalities gaps (SDG 10) we still find in Brazil. Thus, in response to the ongoing syphilis epidemic in Brazil, contributing to the implementation of SDG 10 means “leaving no one behind.” In this context, the learning pathway “Syphilis and other STIs” contributed to implementing this SDG with goals 10.3 and 10.a (55). These goals propose equal opportunities, reduction of inequalities, and the principle of special and differential treatment for developing countries, particularly the least developed countries. In this line, the “Syphilis No” Project reached both key populations (e.g., LBGTQIA+, Men who have Sex with Men, and the Trans Population) and populations considered vulnerable (e.g., prison population) through communication and massive open online health education actions (3, 6, 8).

Online Health Education, as a tool to promote national public policies, is an ethical and conscious action for producing social impacts in cities and communities (SDG 11), as it contributes to rendering them more inclusive, safe, resilient, and sustainable. Creating social impact by implementing SDG 11 also implies valuing the importance of health professionals in communities and, therefore, building and maintaining safe, inclusive, healthier, and more resilient environments. Massive open online health education, when well-directed, can sustainably steer a process of health systems resilience (51, 53), which impacts lives in cities and communities.

The impacts generated by massive open online health education in Brazil—structured through the AVASUS learning pathway—validate the importance of continuing education for health professionals. Through this educational process, we observed not only increased syphilis testing in Brazil, which began to occur more effectively and sustainably (even during the COVID-19 pandemic) but also a drop in CS cases in all Brazilian regions. Therefore, in the context where there are public health crises, it is understood that massive open online health education is a tool for boosting resilience, which can produce social impacts on the public health system, cities, and communities. According to Pinto et al. (3, 6), Rocha et al. (4), and Andrade et al. (1), the SNP has worked as a tool to induce national policies in response to the epidemic, contributing to both the reduction of CS cases and the increase in syphilis testing, diagnosis, and adequate treatment. And these are results that can be measured through epidemiological data analysis. However, the social impacts steered by massive open online health education are transversal, not only to epidemiological data but to the whole society. Therefore, discussions and analysis through the lens of the SDGs can contribute to casting light on social impacts that are often unmeasurable or difficult to measure.

## 5. Conclusion

In view of the importance of developing strategic plans and the necessary foresight for the healthcare system, this study highlighted the importance of massive open online health education during a public health crisis, emergency, or urgency, as was the case of the syphilis epidemic in Brazil. The data-based analyses strongly suggest that the use of MOOCs for health education, as was done in the SNP, could be employed by health authorities as a tool to influence the formulation of public health policy. Our findings underline that, besides comprehensively educating health workers, massive open online health education acts as a tool for promoting resilience in health systems' response to emerging health crises. In this context, managers and other public health authorities should regard this tool as an important element of public health policy strategy.

When analyzing the learning objectives of each course, we observed that the course participants had access to educational resources designed to prepare them to respond to the epidemic considering its various fields and aspects, according to the results and analyses this study presented. In this way, AVASUS courses educated participants from all country regions and amassed hundreds of thousands of registrations from the most diverse healthcare professions and levels of care.

It is worth pointing out the distinctiveness of a universal educational process, as developed by the SNP, not only due to its alignment with the SGDs but also because of the diversity of opportunities for developing transversal work practices through applied training, which favors updated professional education and awareness of one's social role.

This research reveals scientific findings and results from studies conducted by cross-referencing data from various sources, as described in the methodology. When analyzed in combination, such data allowed us to see the impacts of massive open online health education on the epidemiological landscape of syphilis in Brazil. It should be emphasized that this study relied on secondary data. Therefore, future research should apply a model that elicits primary data.

Regarding future prospects, we strongly consider that models for analyzing massive open online continuing education impacts in health services working with primary data based on surveys applied to students could help expand the body of knowledge in the field and the use of current and novel techniques.

## Data availability statement

The datasets presented in this study can be found in online repositories. The names of the repository/repositories and accession number(s) can be found at: https://doi.org/10.5281/zenodo.6549079.

## Author contributions

AC, CG, and RV collaborated in the creation, study design, and performed the analysis. IB and PM processed the data and organized the repository. AC, CG, IB, PM, MR, and RV wrote sections of the manuscript. All authors contributed to the review of the paper and approved the most recent version.

## Funding

This research was funded by Ministry of Health Brazil.

## Conflict of interest

The authors declare that the research was conducted in the absence of any commercial or financial relationships that could be construed as a potential conflict of interest.

## Publisher's note

All claims expressed in this article are solely those of the authors and do not necessarily represent those of their affiliated organizations, or those of the publisher, the editors and the reviewers. Any product that may be evaluated in this article, or claim that may be made by its manufacturer, is not guaranteed or endorsed by the publisher.

## Abbreviations

AVASUS: virtual learning environment of the Brazilian health system
CBO: Brazilian occupation classification
CHAs: community health agents
CHE: continuing health education
CNES: national registry of healthcare facilities
CS: congenital syphilis
FTA-ABS: fluorescent treponemal antibody absorption
LAIS: laboratory for technological innovation in health
LGBTQIA+: lesbian, gay, bisexual, transgender, questioning, queer, intersex, asexual, pansexual, and allies
MoH: ministry of health
MoL: ministry of labor
MOOCs: massive open online courses
MS: maternal syphilis
MSM: men who have sex with men
MTCT: mother-to-child transmission
OER: open educational resources
PAHO: Pan American health organization
PHC: primary health care
PrEP: pre-exposure prophylaxis
SDGs: sustainable development goals
SHC: secondary health care
SINAN: notifiable diseases information system
SNP: syphilis no project
STI: sexually transmitted infection
SUS: Brazilian national health system
THC: tertiary health care
UFRN: Federal University of Rio Grande do Norte
UN: United Nations
UPA: emergency care units
USF: family health centers.
